# Obtaining Cellulose Fibers from Almond Shell by Combining Subcritical Water Extraction and Bleaching with Hydrogen Peroxide

**DOI:** 10.3390/molecules29143284

**Published:** 2024-07-11

**Authors:** Irene Gil-Guillén, Pedro A. V. Freitas, Chelo González-Martínez, Amparo Chiralt

**Affiliations:** Institute of Food Engineering—FoodUPV, Universitat Politècnica de València, 46022 Valencia, Spain; pedvidef@doctor.upv.es (P.A.V.F.); cgonza@tal.upv.es (C.G.-M.); dchiralt@tal.upv.es (A.C.)

**Keywords:** almond shell, valorisation of agri-food waste, subcritical water extraction, hydrogen peroxide, bleaching, cellulose

## Abstract

Almond shell (AS) represents about 33% of the almond fruit, being a cellulose-rich by-product. The use of greener methods for separating cellulose would contribute to better exploitation of this biomass. Subcritical water extraction (SWE) at 160 and 180 °C has been used as a previous treatment to purify cellulose of AS, followed by a bleaching step with hydrogen peroxide (8%) at pH 12. For comparison purposes, bleaching with sodium chlorite of the extraction residues was also studied. The highest extraction temperature promoted the removal of hemicellulose and the subsequent delignification during the bleaching step. After bleaching with hydrogen peroxide, the AS particles had a cellulose content of 71 and 78%, with crystallinity index of 50 and 62%, respectively, for those treated at 160 and 180 °C. The use of sodium chlorite as bleaching agent improved the cellulose purification and crystallinity index. Nevertheless, cellulose obtained by both bleaching treatments could be useful for different applications. Therefore, SWE represents a promising green technique to improve the bleaching sensitivity of lignocellulosic residues, such as AS, allowing for a great reduction in chemicals in the cellulose purification processes.

## 1. Introduction

Almond is the most produced nut worldwide in recent years. The almond tree (*Prunus dulcis*) is characterised by being cultivated in arid areas with dry conditions, thus motivating the establishment of sustainable irrigation practises to ensure high production [1]. Thus, the worldwide almond production was of 1.5 Mt in 2021 [2]. The world’s leading producer of almonds is the United States, with about 1.1 Mt per year [2]. Almond fruit consists of four portions: kernel or meat (~11%), middle shell (~33%), outer green shell cover or almond hull (~52%), and a thin leathery layer known as brown skin (~4) [3]. The almond shells and hulls account for more than 70% of the dry weight of the almond fruits, which have been mainly used as animal feed or burned for fuel production and energy use. Almond shell is the lignocellulosic material forming the husk of the almond fruit with very reduced value and potential environmental problems. Its valorisation is interesting, providing a second income from the crop and producing value-added compounds, contributing to the circular economy. The almond variety, environmental factors, and agronomic practises have a significant influence on the composition of the almond components, determining the final physicochemical and phytochemical properties of both the kernel and its by-products [4]. The main constituents of almond shells are cellulose, hemicellulose, and lignin, and their contents vary between 20 and 38%, 8 and 28% and 20 and 50%, respectively [5,6,7]. Based on this composition, Morales et al. [8] have proposed a multiproduct biorefinery approach to obtain hemicellulosic fractions, high-purity lignin, and cellulose nanocrystals from ASs in order to valorise this by-product. Other studies have also reported different applications of AS, such as obtaining activated carbon [9], low-cost adsorbent for contaminated solutions [10,11], or reinforcing materials in different polymeric matrices, such as polypropylene [12], polylactic acid [13], or starch [14].

Numerous studies have focused on the extraction and application of natural fibres from plant materials to obtain polymer composites useful in developing materials with more sustainable characteristics [15,16] and, specifically, for food packaging applications [17,18,19]. The natural origin of the fibres causes, in general, a wide range of variations in properties depending on several factors, such as the harvesting location and conditions. The demand for natural fibres has recently increased because of their low cost, biodegradability and eco-friendliness [19]. Peças et al. [15] have published a comprehensive review about the properties of natural fibres used as reinforcement in composite materials and their potential applications.

Several studies have been carried out on the production of cellulose from almond shells. Alhaji Mohammed et al. [20] studied the effect of particle size and extraction parameters on the purity and yield of nanocellulose, using solvent extraction, alkaline hydrolysis, and bleaching with sodium chlorite. These authors also analysed the effect of acid hydrolysis parameters (acid type, acid concentration, reaction time, and temperature) on the properties of nanocellulose extracted from almond shells [21]. Nanocellulose from ASs was also obtained by dewaxing–alkali treatment–bleaching with subsequent hydrolysis with sulphuric acid [22,23]. Pulping methods using different reagents, including alkaline, acid, and organosolv solutions, have been used for delignification of ASs in order to obtain cellulose nanopaper [6]. Cellulose nanofibers have also been obtained from ASs by applying alkaline and sodium chlorite treatments for delignification and TEMPO-oxidising [24]. Maaloul et al. [25,26] used ionic liquids (1-butyl-3-methylimidazolium chloride) to dissolve cellulose from ASs and their separation for application as a copper (II) absorber from aqueous solutions. To optimise the use of chemicals and time processing for cellulose production, Valdes et al. [27] applied microwave-assisted extraction of cellulose from AS. However, the recovery of compounds from agri-food waste using more sustainable extraction practises is necessary due to environmental and economic aspects [28]. In this sense, no previous studies were found to apply subcritical water extraction (SWE) to promote cellulose extraction from AS.

Subcritical water extraction (SWE) is proposed as an environmentally friendly and economically viable alternative to conventional extraction processes, such as solvent extraction. This green extraction technique uses water as the extracting agent, as opposed to conventional extraction procedures such as the solid–liquid (Soxhlet) process using organic solvents. SWE demonstrates superior yield while shortening extraction time [29,30]. In the SWE process, the feedstock is heated in the aqueous phase at subcritical temperature (150–320 °C) and pressure (20–150 bar). Under these conditions, the properties of water, such as dielectric constant, surface tension or viscosity, are modified, facilitating mass transfer and the extraction of poorly water-soluble compounds. This is also favoured by the fact that subcritical water drives the hydrolysis of certain bonds in the agri-food waste matrix, releasing bonded compounds to the aqueous media [31]. Multiple applications of SWE have been studied in the extraction of bioactive compounds from agri-food waste, including stilbenes from vine co-products [32], phenolic compounds from onion peel [29], or protein hydrolysates from seafood waste [33], among others.

On the other hand, increasing concern about the environmental consequences of bleaching processes using chlorine and its derivatives has led to the exploration of bleaching techniques based on chlorine-free compounds [34,35]. These bleaching procedures are based on oxygenated compounds, with hydrogen peroxide being a bleaching product that has proven to be highly efficient and competitive in terms of ability to promote discolouration, reducing costs and minimising ecological impact [35,36]. In recent studies, hydrogen peroxide has been used as a bleaching agent, in combination with previous water extraction at subcritical conditions, for the purification of cellulose from almond skins [37] and rice straw [38], with promising results.

Considering the high and selective extraction capacity of SWE, and its ability to hydrolyse hemicellulose and other non-cellulosic constituents [30,31], it could be used to partially purify cellulose in lignocellulosic biomass waste in order to obtain cellulose fibres. Nevertheless, the use of SWE to remove non-cellulosic components from lignocellulosic residues has been largely unexplored. In a recent study [39], SWE was used as a pre-treatment of pulping and bleaching steps for isolation of cellulose fibres from wetland reed grass, promoting a higher yield of cellulose with less severe conditions of the pulping and bleaching process steps. Likewise, cellulose fibres from rice straw were isolated by combining SWE and bleaching with sodium chlorite, with good yield and purity [40].

The novelty of the present study is based on the use of SWE as a green extraction technique, reducing the use of chemicals, in combination with a greener bleaching method with hydrogen peroxide to obtain cellulose fibres from almond shells. Thus, subcritical water extraction at two temperatures was applied as a first treatment to remove non-cellulosic compounds from the AS powder. Afterwards, the obtained lignocellulosic residues were submitted to a bleaching treatment using hydrogen peroxide at pH 12 in consecutives 1 h cycles. Purified cellulose fractions were analysed as to their yield, whiteness index, and composition. The effectiveness of the bleaching step purification was compared to that obtained with sodium chlorite. The obtained fibres were also characterised by their microstructure, crystallinity, and thermal stability.

## 2. Results and Discussion

### 2.1. Yield and Composition of SWE Solid Fractions from Almond Shell

**Figure 1:**
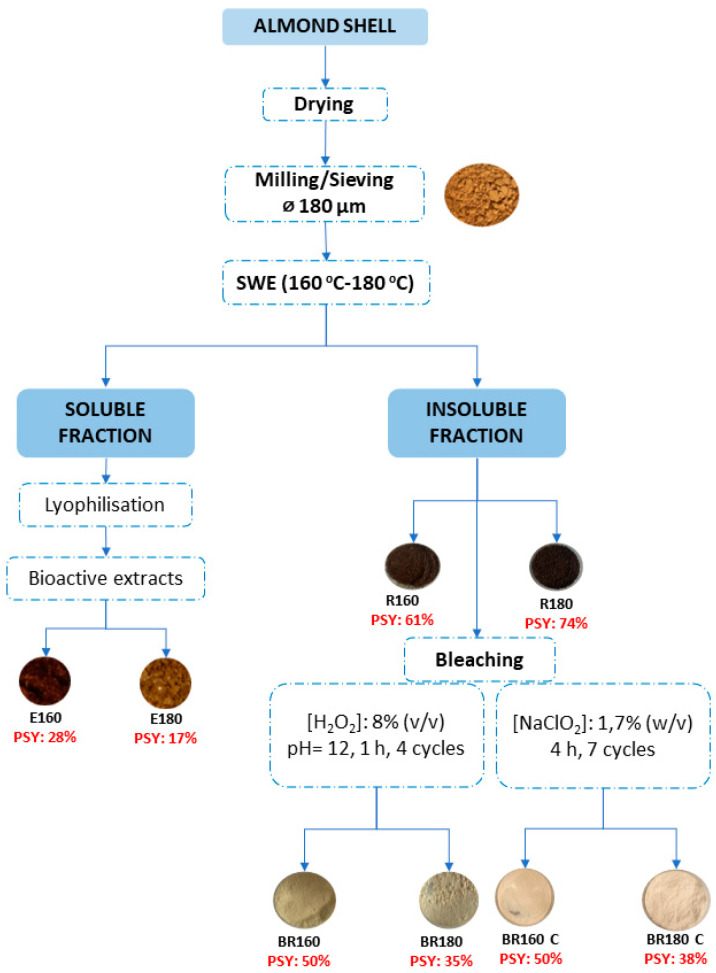
Flow chart diagram of the process used to obtain cellulose from almond shell, indicating the mass yields (mass of outcome solids/mass of income solids, in percentage) of each process step.

Figure 1 shows the flow chart of the applied process to obtain cellulose from almond shells. Ground almond shells (<180 μm) were initially submitted to subcritical water extraction to remove as many non-cellulosic compounds as possible without using chemicals, taking advantage of the high extractive power of water under subcritical conditions. Two temperatures were tested in a range reported in previous studies to allow for a good extraction of phenolic compounds and hemicellulose [41,42]. A good mass extraction yield (28 and 17% of dry raw material, respectively, at 160 and 180 °C) was obtained, giving rise to lignocellulosic residues (R160 and R180) that represented 61 and 74% of the initial almond shell. The respective sums of the yields in extract solids and insoluble residues at each temperature indicate that only 89–91% of solid matter was recovered in the process, which could indicate a partial degradation of organic compounds during this treatment at high temperature, as observed in previous studies for almond skin under similar conditions [37]. The composition of solid residues (R160 and R180), in comparison with the initial AS, is shown in Table 1, where protein, structural polymers, and ashes of these materials are reflected. The solid residues were clearly enriched in cellulose, compared to the raw material, due to the partial removal of lignin, hemicellulose, and protein during the SWE treatment.

The content of structural polymers in ASs was in the range of previously reported values for other almond varieties [5,6,7]. A part of the cellulose richness (27%), the AS sample was also rich in hemicellulose (24%) and lignin (21%), with very low contents of protein and ashes. After the SWE process, the solubilisation of different compounds gave rise to a significant increase in the cellulose content of the solid fractions (42–45% in R160 and R180), with a relatively small reduction in hemicellulose and lignin. The treatment at 180 °C was more effective at removing hemicellulose from ASs than that carried out at 160 °C. The selective extraction of hemicellulose has been reported to be temperature dependent in previous studies, depending on the plant substrate [42]. No remarkable differences in cellulose content were observed for both solid residues, but the different extraction conditions could have affected the structure of the lignocellulosic complex to a different extent, making it more or less sensitive to the subsequent bleaching treatment.

The values of the water extractives were 16.3 ± 1.5, 18 ± 3, and 24 ± 6%, respectively, for the AS, R160, and R180 samples, while much lower values were obtained in ethanol (1.6 ± 0.5, 3.7 ± 0.7, and 7.1 ± 1.33, respectively, for AS, R160, and R180 samples). These values indicate that soluble solids remained in the extraction residues (R160 and R180), probably due to the hydrolytic effect of SWE that promotes the release of initially bonded compounds into the plan matrix, as observed in other plant products [37,40].

### 2.2. Yield and Composition of Bleached Cellulosic Fractions

**Figure 2:**
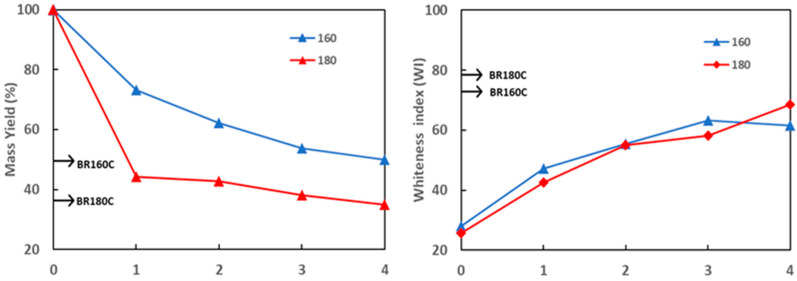
Development of bleaching yield (BY) and whiteness index (WI) of the lignocellulosic solids obtained from SWE at 160 and 180 °C throughout the bleaching cycles with hydrogen peroxide (BR160 and BR180). The values of BY and WI obtained by bleaching with sodium chlorite (BR160C and BR180C) are indicated on the Y-axis of the plot.

Bleaching treatment was applied in four cycles with hydrogen peroxide at 8% (*v*/*v*), at pH 12, as previously optimised for almond skin [37] and rice straw [38]. The progress of sample bleaching was monitored through the development of the mass bleaching yield (BY), which quantifies the removal of non-cellulosic compounds, and the whiteness index (WI), which indicates the removal of coloured compounds. Figure 2 shows the development of BY and WY of the samples R160 and R180 throughout the different cycles. A higher decrease in BY was already observed for the R180 sample during the first cycle, reaching 35% yield in the last cycle. However, the WI was slightly lower in sample R180 than in R160 throughout the different cycles, except in the last four cycle when it reached higher whiteness. These results indicate that the removal of compounds by the effect of the bleaching agent occurred to a greater extent in sample R180, although coloured compounds in this sample were more recalcitrant and disappeared more slowly. Apart from the original-coloured compounds in the AS, other brownish compounds could be formed during the SWE step, especially at the highest temperature. Plaza et al. [43,44] reported the formation of caramelisation and Maillard compounds under subcritical water extraction, which would contribute to the more intense colour of samples R160 and R180, compared to the initial AS powder (Figure 1). These neo-formed coloured compounds could be more resistant to bleaching in sample R180, which could explain its slightly greater resistance to bleach.

The BY and WI of the R160 and R180 samples bleached with sodium chlorite (seven cycles) were also shown in Figure 2 for comparison purposes. Similar BY values to those obtained with hydrogen peroxide were obtained for BR160C and BR180C samples, but with higher WI values (72 and 79, respectively, for R160C and R180C), thus indicating the greater removal of coloured compounds from both lignocellulosic residues with sodium chlorite. The bleaching process was also slightly more effective in sample R180 than in R160, as observed for the treatment with hydrogen peroxide with four cycles.

To compare the cellulose purity reached in the R160 and R180 samples after bleaching with both agents, Table 1 shows the contents in the different structural components analysed in all bleached samples. The reached cellulose contents ranged between 70 and 83%, depending on the extraction residue and bleaching agent. Sodium chlorite gave rise to a slightly higher content of cellulose, and the extraction residue R180 tended to be richer in cellulose for both bleaching treatments, although considering the variability no significant differences were found for most of the cases. As concerns lignin content, the R180 sample was more sensitive to delignification by hydrogen peroxide than R160. In contrast, the opposite effect was observed with sodium chlorite. The different mechanisms involved in the bleaching reactions with hydrogen peroxide and sodium chloride, as well as the different accessibilities of reactants to the target points in the plan matrix, would be responsible for the observed differences. It is remarkable that hemicellulose was also not quantitatively removed in the bleaching treatments and bleached samples maintained similar contents to the corresponding extraction residues (20 and 12% for R160 and R180 samples, respectively).

These results indicate the difficulty of cellulose purification in ASs using hydrogen peroxide or sodium chlorite, but SWE at the highest temperature facilitates this process by removing more hemicellulose from the matrix and promoting delignification during the bleaching step. Thus, at the end of the process, the BR160 and BR180 samples represented 31 and 26%, respectively, of the initial dry AS, with a cellulose purity of 71 and 78%, respectively. This cellulose content referring to initial AS dry mass corresponds to 21.5 and 20.3 g cellulose/100 g AS, respectively. Therefore, taking the initial cellulose content into account (27%), a loss of cellulose (6–7 g/100 g AS) occurred throughout the bleaching process, probably due to its oxidation by free radical mechanisms with hydrogen peroxide, as described by other authors [45]. The cellulose contents of the BR160C and BR180C samples, treated with sodium chlorite, were in the same range (77–84% in both residues), but when referred per mass unit of dry AS, they were 23–24 g/100 g AS; these values are closer to those determined in the initial AS, which suggests lower losses of cellulose during the process.

### 2.3. FTIR Spectroscopy Analysis

**Figure 3:**
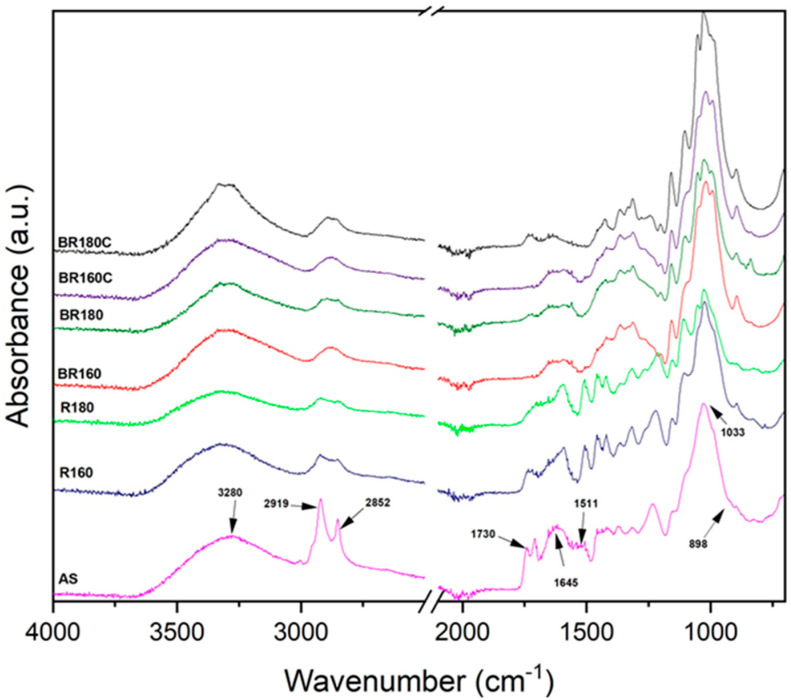
Comparison of FTIR spectra of lignocellulosic samples from almond skin (AS) after the SWE process at 160 °C (R160) and 180 °C (R180) and after the bleaching treatment with hydrogen peroxide (BR160 and BR180) and with sodium chlorite (BR160C and BR180C).

Figure 3 shows the FTIR spectra of the untreated raw material, the extraction residues (R160 and R180), and the bleached samples with H_2_O_2_ (BR160 and BR180) and sodium chlorite (BR160C and BR180C). All the samples exhibited a broad, bell-shaped absorption band between 3000 and 3800 cm^−1^, which is related to the −OH stretching vibration. This band gained relative intensity and sharpness as the cellulose purification progressed during the SWE and bleaching processes due to the progressive removal of amorphous components such as hemicellulose and lignin [46]. The absorption band at 2919 cm^−1^ was due to the aliphatic saturated C-H stretching vibration in cellulose and hemicelluloses, while the vibration band at 2852 cm^−1^, originating from C-H stretching in lignin and waxes, was highly attenuated after the different purification treatments, with respect to the initial AS samples. The band at 1730 cm^−1^ corresponds to the stretching vibration of the carbonyl group (C=O) in the phenolic and uronic acids, present in the lignin and hemicellulose fraction, respectively [47]. The intensity of this peak decreased after the SWE process, especially in the R180 sample, and practically disappeared after the bleaching treatments in the BR160, BR180, BR160C, and BR180C spectra. The narrow peak at 1511 cm^−1^ corresponds to the C=C double bond stretching vibration of the aromatic skeletons present in the lignin rings [48]. This absorption band is visible in the AS raw material and the extraction residues R160 and R180, but it is not appreciable in bleached samples, thus showing the effectiveness of the bleaching treatments. The absorption band at 1033 cm^−1^ corresponding to the stretching vibration of the C-O-C-O-C bond of the acetal bond present in cellulose and hemicellulose stands out. This band grows in relative intensity when the cellulose content increases, reaching its maximum relative value for BR180C, according to the highest content of cellulose. The absorption band at 898 cm^−1^ is related to the stretching vibration of the β-glycosidic bond of cellulose [40], and its relative intensity increased highly in bleached samples. Therefore, the FTIR spectra also show the efficiency of the combined SWE and bleaching treatments for the purification process of cellulose from almond shell.

### 2.4. Thermogravimetric Analysis

**Figure 4:**
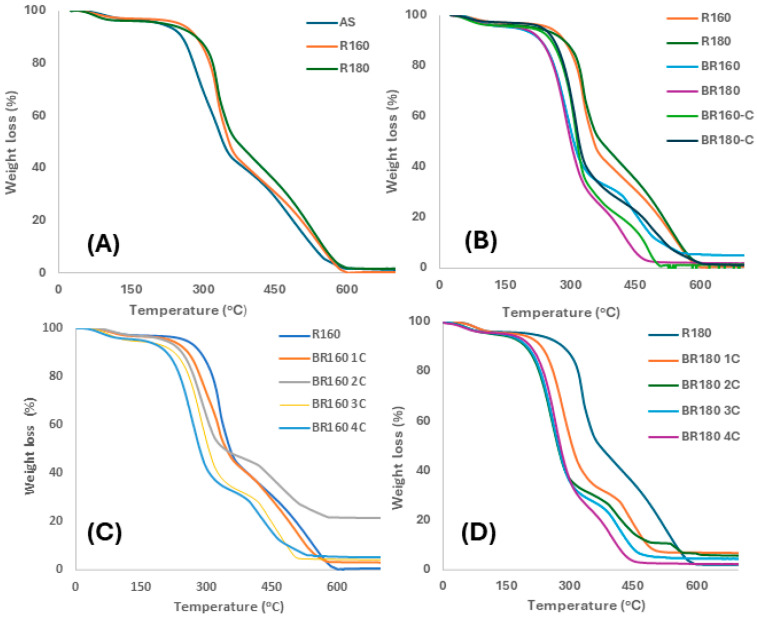
TGA curves of the different lignocellulosic residues at the different process steps. (**A**) ASs and extraction residues R160 and R180. (**B**) The extraction residues R160 and R180 before and after bleaching with hydrogen peroxide (BR160 and BR180) and sodium chlorite (BR160C and BR180C). (**C**,**D**) Samples R160 and R180 before and after the successive bleaching cycles with hydrogen peroxide.

Thermal stability of the lignocellulosic residues was analysed as an indicator of the cellulose quality obtained in the process. Figure 4 shows the TGA curves of the different samples at the different process steps, for comparison purposes. In every case, the typical weight loss steps of lignocellulosic samples can be observed, with changes associated with the different compound removals in each step. The first weight loss step (about 5% wt.) before 100 °C corresponds to the loss of bound water in the samples, as also observed by other authors [6,7]. The TGA curve of the initial ASs shows the typical shape previously reported [6,7,22], with an early weight loss around 287 °C (temperature peak in DGTA curve) and a second weight loss at 350 °C (temperature peak in DGTA curve), with similar weight loss associated with each peak. The mass loss at 200–400 °C is mainly related to the depolymerization of hemicellulose and the cleavage of the glycosidic linkage of cellulose [49], while the mass loss at 420–600 °C can be mainly attributed to the decomposition of the lignin fraction. Modica et al. [7] observed that pure lignin isolated from ASs exhibited two mass loss steps in the ranges of 200–400 °C and 420–600 °C, while pure cellulose isolated from ASs exhibited a main great loss in the range of 200−400 °C.

Figure 4A shows the TGA curves of ASs and the extraction residues (R160 and R180), where the above-described behaviour was observed between 200 and 600 °C, associated with the degradation of hemicellulose, cellulose, and lignin. The temperature of the maximum degradation rate in the first main step was higher for the extraction residues R160 and R180 (330 °C), of which TGA curves practically overlapped coherently with the removal of low molecular weight compounds and the increase in cellulose content similarly in both samples. In the bleached samples with hydrogen peroxide and sodium chlorite (Figure 4B), a sharper and more intense weight loss could be observed in the first degradation step, coherent with the increase in the cellulose content, in comparison with the non-bleached R160 y R180 samples. The different cellulose purification degrees can be appreciated in the curves, where samples BR160 and BR180C reflect their higher lignin content (Table 1).

The bleached samples treated with hydrogen peroxide exhibited a lower temperature of the maximum degradation rate of cellulose than those treated with sodium chlorite (300 °C vs. 315 °C). This lower temperature suggests the greater partial degradation of cellulose, already deduced from mass balance, by oxidative mechanisms with hydrogen peroxide, giving rise to less thermostable shorter chains. Vismara et al. [45] described the mechanisms of cellulose oxidation with hydrogen peroxide through the formation of alpha hydroxyalkyl radicals and subsequent chain scission, forming end chain carbonyl groups. The TGA curves of the samples treated in different cycles with hydrogen peroxide also revealed the partial cellulose degradation (Figure 4C,D). The progressive shift of the cellulose degradation temperature towards lower values can be clearly observed for the R160 and R180 samples as the cycles progress. Nevertheless, this development was delayed in the R160 sample with respect to the R180 samples, of which TGA curves practically overlapped from the second cycle onwards. The progressive sample delignification can also be observed in Figure 4C,D through the reduction in the lignin-associated degradation step. The greater accessibility of the bleaching agent to target points in the R180 sample, probably due to the higher hemicellulose removal during the SWE step, could explain the different bleaching behaviours of R160 and R180, as also observed in the different progressions of the bleaching yield (Figure 2).

Therefore, the thermal analyses reflect the different effects of the bleaching agents on the cellulose properties, the effect of SWE temperature on the bleaching rate with hydrogen peroxide, and the difficulty of completely purifying cellulose from AS, as also observed by other authors, even using the usual alkaline treatments [6,7]. 

### 2.5. Crystallinity Analysis

**Figure 5:**
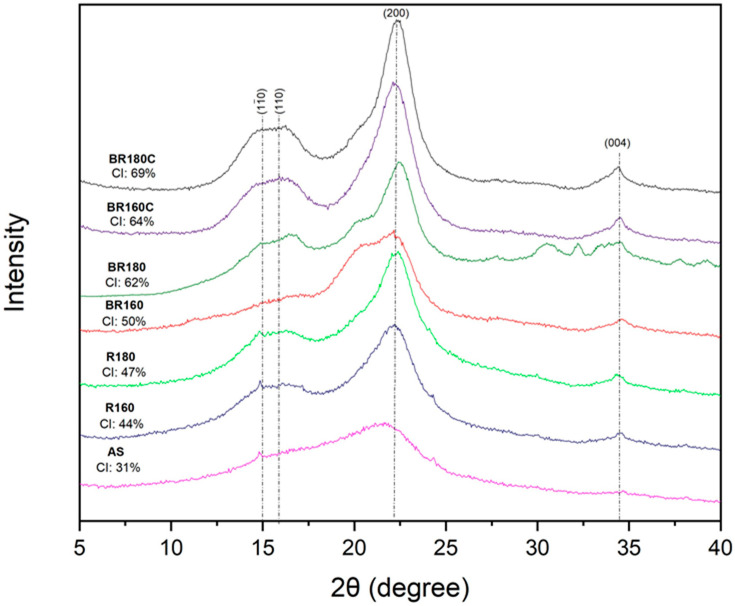
X-ray diffractograms and the crystallinity index (CI) of the different lignocellulosic residues: ASs, SWE solid residues (R160 and R180), and bleached samples with hydrogen peroxide (BR160 and BR180) and sodium chlorite (BR160C and BR180C).

Figure 5 shows the X-ray diffraction patterns and the crystallinity index (CI) of the raw material (AS), the extraction residues (R160 and R180), and the cellulosic fractions obtained in the bleaching process with hydrogen peroxide (BR160 and BR180) and sodium chlorite (BR160C and BR180C). The typical diffraction peaks of the crystalline structure of cellulose type I at 2θ values of 16° (110), 22° (200) and 34° (004) [50] were observed for every sample, with different resolutions depending on the cellulose purity. Overall, after the bleaching processes, the crystalline peaks strengthened coherently with the removal of amorphous components, such as lignin and hemicellulose, and the enrichment in cellulose [51]. The values obtained for CI in the different samples were consistent with the compositional data and the FTIR spectra. The percentage of crystallinity already increased after the SWE process according to the release of a great part of amorphous material. The sample R180 exhibited a higher crystallinity index than R160, coherent with the greater extraction of hemicellulose, as shown in Table 1. The bleaching treatments and their delignification effect also implied an increase in the sample crystallinity, with this being higher in the samples treated with sodium chlorite, which ratifies their higher cellulose richness. The combined treatment of SWE at 180 °C and bleaching with sodium chlorite (sample BR180C) gave rise to the highest crystallinity index (69%). This value was higher than that reported by other authors for cellulose extracted from AS using alkaline treatment, bleaching with sodium hypochlorite, and sulfuric acid hydrolysis (67.5%) [23]. Rashid et al. [22] obtained values for CI of 52.45% for raw almond shell, increasing to 68.21% when submitted to alkaline extraction and bleaching with sodium hypochlorite containing NaOH and glacial acetic acid. Other authors [6] obtained values of CI slightly higher for AS cellulose before and after KOH pulping and bleaching (59.17 and 72.68%, respectively). The different values on the crystallinity index in raw ASs are related to different cellulose contents, which would also affect the purification effectiveness. 

It is remarkable that in both the H_2_O_2_-bleached samples (BR160 and BR180), the diffraction peak (200) exhibited a shoulder at about 20° that did not appear in the other bleached samples. This could be attributed to the partial formation of cellulose II, with diffraction peaks at 2θ = 12.2° ($1\bar{1}0),\;$2θ = 19.89° (110), and 2θ = 21.96° (200) [52]. Changes in the allomorphic form of cellulose can be caused by alkaline treatments with NaOH at different concentrations when Na ions are incorporated into the crystal lattice, producing antiparallel Na–cellulose crystalline complexes that alter the crystallinity of the material, thus contributing to the remodelling of cellulose microfibrils [53]. During bleaching treatment with hydrogen peroxide containing NaOH (0.04%), where cellulose partially depolymerised, as deduced from TGA, the partial rearrangement of cellulose crystalline regions could have also occurred, giving rise to cellulose II crystalline formations. The maximum diffraction peak of these formations would appear to overlap with those of cellulose I, giving rise to the shoulder observed in the spectra at 2θ = 22°. Therefore, the bleaching agent affected the final properties of the AS cellulose, partially modifying its thermal and crystallisation pattern.

### 2.6. Morphological Analysis

**Figure 6:**
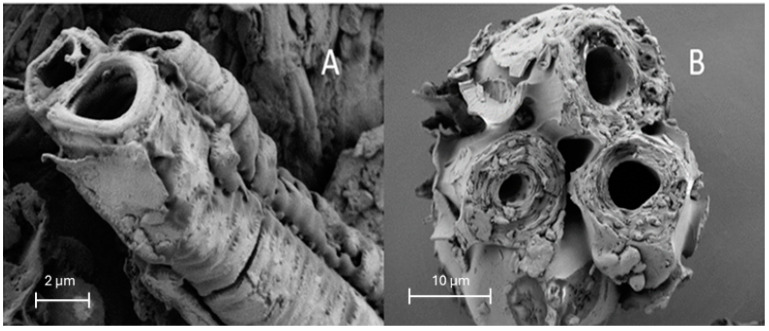
FESEM images of two kinds of particles present in the ground AS. (**A**) From the vascular bundles, (**B**) from the inner or outer shell.

Almond shell has a three-layer structure consisting of inner and outer shells, connected by an intermediate layer of vascular bundles, with all of these containing cellulose in different arrangements. Modica et al. [7] identifies two kinds of cellulosic particles in the cellulosic fraction of AS: cellulose fibres of the secondary cell wall of spiralled tracheas, constituting the vascular bundles, and micro-cellulose particles from the inner and outer shells. In these layers, a pore structure has been described, with a row of large holes distributed in the cross-section and dense material around the holes [5]. Figure 6 shows two representative particles of these types of structures found in the ground ASs when observed by FESEM. The almond shell appears to be composed of tightly compacted hollow cylindrical formations of 40–50 μm diameter, with a cylinder wall thickness of about 10 μm. Tiny holes can be appreciated on the cylinder wall, which is clearly layered, as previously reported by Li et al. [5]. Therefore, ASs have a highly porous structure that provide them with excellent properties as an adsorbent material, as observed in different studies [9,10,22].

**Figure 7:**
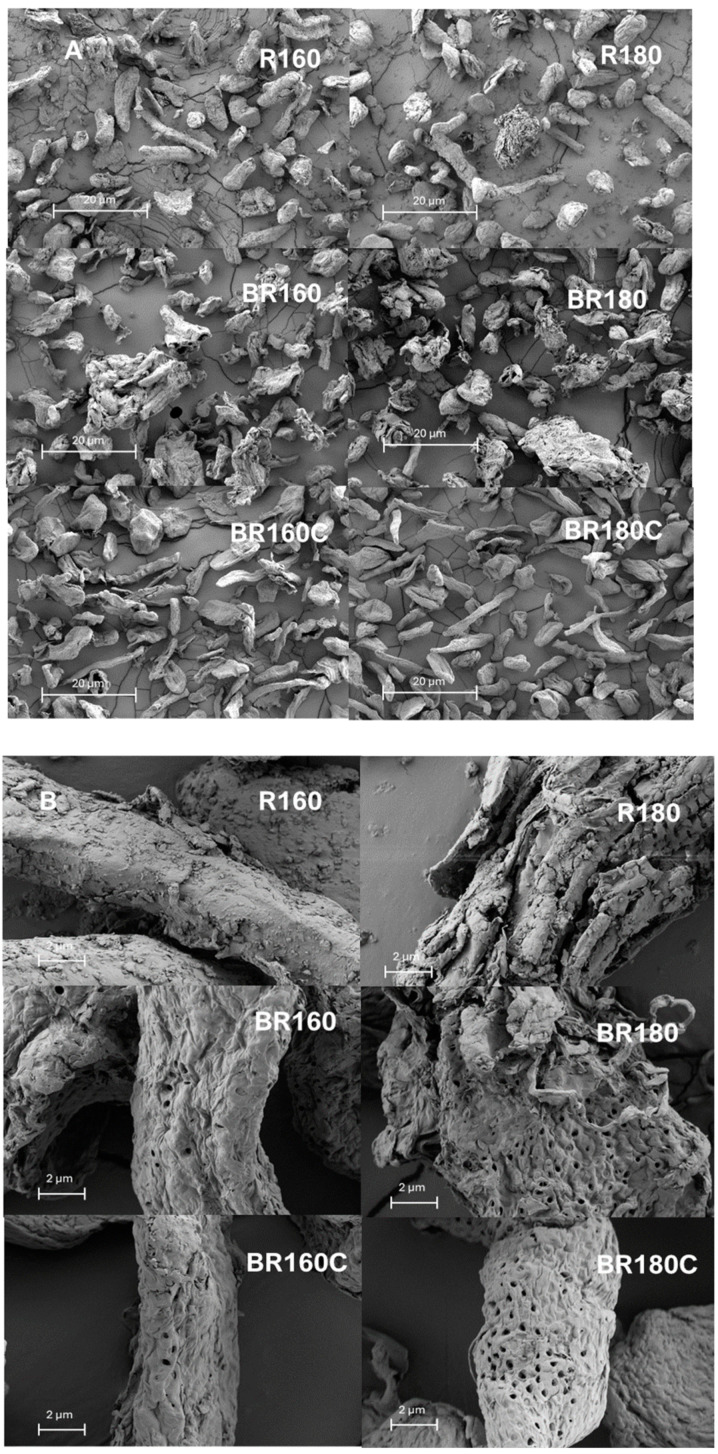
HRFSEM images ((**A**): 200X and (**B**): 2000X) of the different lignocellulosic residues: AS, SWE residues (R160 and R180) and bleached samples with hydrogen peroxide (BR160 and BR180) and sodium chlorite (BR160C and BR180C).

Figure 7A,B shows, at different magnifications, the morphology of representative lignocellulosic particles of the SWE-treated samples at 160 and 180 °C (R160 and R180), and their respective bleached samples with hydrogen peroxide (BR160 and BR180) and sodium chlorite (BR160C and BR180C). Predominantly enlarged particles can be observed in the R160 and R180 samples, while these appeared more distorted after bleaching with hydrogen peroxide, and thinner and longer after bleaching with sodium chlorite. This is in agreement with the alteration provoked in the cellulose by hydrogen peroxide, as was previously commented on. At higher magnification, in Figure 7B, the surface microstructure of cellulosic particles can be better appreciated. This considerably changed throughout the SWE process and bleaching step with the different agents. The particles of the R160 and R180 samples showed different alterations depending on the extraction temperature. At the highest temperature (180 °C), particles exhibited a more cracked surface, revealing the more intense rupture of the tissue in line with the removal of different compounds. After the bleaching process, a cleaner particle surface can be observed, while the porous structure of the cellulose arrangement can also be appreciated. Other authors [21,22] have also described the porous cellulose structure at different structural levels, even in nanocellulose material. Rashid et al. [22] reported pitted and porous monolithic structures for AS cellulose with pore sizes ranging between 0.8 μm to 2.0 μm and very few rod-like structures, but this was not observed in other studies [23]. The appearance of the bleached sample with sodium chloride was smoother and less distorted than that observed for samples treated with hydrogen peroxide, according to the different degrees of cellulose purification and/or alteration. Microstructural differences in the cellulose particles could impact their performance in potential applications, such as the development of cellulose films or aerogels, reinforcing the effect on composites or productions of drug delivery systems or binders in pharmaceutical applications. Therefore, further studies are required to elucidate the key properties of the fibres, such as aspect ratio and reinforcing capacity, interfacial adhesion with other materials, water and oil absorption capacities, or adsorption/release capacity of different target compounds.

## 3. Materials and Methods

### 3.1. Materials

The middle almond shells (ASs) (var. Guara) were kindly supplied by Importaco SA (Valencia, Spain) from their 2022 harvest. A forced air oven (SP Selecta, sa, Barcelona, Spain) was used to dry the sample for 3 days at 40 °C. The dry sample was then ground with a mill (Model SM300 stainless steel, Retsch GmbH, Haan, Germany), and the obtained powdered material was sieved to a particle size of less than 180 μm.

Sodium chlorite, sodium hydroxide (NaOH), arabinose, and glucose were provided by Sigma-Aldrich (St. Louis, MO, USA). Glycerol, sulphuric acid (H_2_SO_4_, 98%), hydrogen peroxide (H_2_O_2_, 30%), sodium carbonate (Na_2_CO_3_, 99,5%), and phosphorus pentoxide (P_2_O_5_) were all obtained from Panreac Quimica S.L.U (Castellar del Vallés, Barcelona, Spain). Sodium acetate trihydrate was supplied by Fluka TM (Stein-heim, Germany). D (+)- Xylose was obtained from Merck KGaA (Darmstadt, Germany).

### 3.2. Fractionation of ASs by Applying Subcritical Water Extraction (SWE)

Almond shell powder was submitted to the subcritical water extraction (SWE) process in a pressure reactor (Model 1-TAP-CE, 5 L capacity, Amar Equipment PVT. LTD, Mumbai, India). A 1:6 (*w*/*w*), AS/distilled water ratio was used at two extraction temperatures (pressures), 160 °C (7 bar) and 180 °C (15 bar, with 150 rpm stirring, for 30 min). From each extraction step, soluble extract and insoluble residue were separated by vacuum filtration. The insoluble residues obtained (R160 and R180) were washed with distilled water, dried in a forced air stove at 40 °C for 2 days, and stored at 5 °C until use. The dry mass yields of the R160 and R180 samples were determined with respect to the initial dry mass of the AS. The extraction yields were also determined by sampling two aliquots of the liquid extracts and drying at 105 °C until constant weight. Thus, the solid/water ratio was determined, and the total solids extracted from ASs was calculated, taking the total water mass in the reactor into account.

### 3.3. Bleaching Process of the Extraction Residues

Both extraction residues, R160 and R180, were submitted to the bleaching process to purify cellulose. To this end, a procedure previously applied to almond peel [37], adapted from another study [54], was used, with 8% (*v*/*v*) hydrogen peroxide. The reaction temperature and pH (fitted with NaOH) were kept constant at 40 °C and 12, respectively, using a bleaching solution/solid ratio of 30:1. Four consecutive 1 h cycles were applied, filtering and washing the solid with distilled water. The final cellulosic solids were dried for two days at room temperature, thus obtaining the bleached samples BR160 and BR180 with different cycles applied. To check the efficacy of the bleaching process, the samples were analysed as to mass yield after each cycle and the whiteness index (WI), calculated by applying Equation (1), where the colour coordinates a* (red–green), b* (yellow–blue), and L* (lightness) were measured in the sample powders using a CM-3600d spectrocolourimeter (Minolta Co., Tokyo, Japan), considering illuminant D65 and observer 2°.

(1) $$WI=100-\sqrt{(100-{L*)}^{2}+a^{*2}+b^{*2}}$$

For comparison purposes, the samples R160 and R180 were also bleached with sodium chlorite. The bleach solution was prepared by combining equal parts of sodium chlorite (1.7% *w*/*v*), distilled water, and acetate buffer 2 N (pH = 4.5), according to previous studies [38]. The extraction residues were then heated under reflux together with the bleaching solution (5% *w*/*v*) for 4 h. The dispersion was then washed with plenty of distilled water, filtered, and dried for 1 day at 40 °C, yielding the samples BR160C and BR180C. For each sample, the bleaching process was repeated seven times to reach enough whiteness. The final bleaching yield and WI were also determined in these samples.

### 3.4. Characterisation of Lignocellulosic Fractions

#### 3.4.1. Chemical Composition

The contents of the structural components (cellulose, hemicellulose, and lignin), protein, and ashes of the almond shells (ASs), extraction residues (R160 and R180), and bleached samples (BR160, BR180, BR160C, and BR180C) were determined. Prior to the quantification of structural components, the extractives were analysed, as described by the NREL standard method (NREL/TP-510-42619-2008) [55]. In the first step, water extraction was carried out, followed by an extraction with ethanol, both for 6 h, using a Soxhlet apparatus. Structural carbohydrates and lignin were determined according to the NREL standard method (NREL/TP-510-42618-2008) [56] in the extractive-free samples. This process consisted of a two-stage acid hydrolysis of about 300 mg of sample: (1) with 3.0 mL H_2_SO_4_ at 72%, for 1 h, at 30 °C, and (2) after dilution with 84 mL of deionised water, followed by autoclaving at 121 °C, for 1 h. From the acid insoluble fractions, the Klason lignin content was gravimetrically quantified. The soluble fractions were used to determine the monosaccharide composition (arabinose, xylose, and glucose) using high performance liquid chromatography (HPLC, Agilent Technologies, model 1120 Compact LC, Waldbronn, Germany). A RezexTM RCM-Monosaccharide Ca^2+^ column (150 × 7.8 mm) and an evaporative light scattering detector (ELSD Agilent Technologies 1200 Series, Waldbronn, Germany) were used. The detector conditions were as follows: gain 3, 40 °C, and 3 bar N_2_ pressure. The mobile phase was deionised water, at a flow rate of 0.4 mL.min^−1^, in isocratic mode. ChemStation software (version LTS 01.11, Agilent Technologies, Waldbronn, Germany) was used in the analysis, while the data were analysed using Origin software (version OriginPro 2021, OriginLab Corporation, Northampton, MA, USA), using the Gaussian model for the determination of the area under the peak. The amount of cellulose was determined from the glucose content, while the amount of hemicellulose was determined from the levels of xylose and arabinose [56]. Each sample was analysed in triplicate.

The ash contents of the samples were determined in triplicate by incineration at 575 °C for 24 h. The protein content was determined by using the Dumas combustion method (Leco, St. Joseph, MI, USA), in duplicate. A conversion factor 6.25 was applied to estimate the protein content from total nitrogen.

#### 3.4.2. Fourier Transformed Infrared (FTIR) Spectra

The FTIR spectra of the different lignocellulosic samples were obtained in triplicate for each sample, using an FTIR spectrometer (Agilent Cary 630 FTIR Spectrometer), equipped with an attenuated total reflectance accessory, in the wavelength range of 4000–650 cm^−1^, at a resolution of 6 cm^−1^, and 128 scans for each spectrum.

#### 3.4.3. Thermogravimetric Analysis

The thermal stability of the different samples, previously conditioned for 1 week at 25 °C in desiccators with P2O5, was determined using a thermogravimetric analyser (TGA 1 Stare System, Mettler-Toledo, Greifensee, Switzerland). The samples (3–4 mg) were weighed in alumina crucibles and heated from 25 °C to 900 °C at 10 °C.min ^−1^, under nitrogen flow (10 mL.min ^−1^). The TGA and DTGA curves were obtained and analysed with STARe evaluation software (version V12.00a, Mettler-Toledo, Inc., Greifensee, Switzerland). The analysis was performed in duplicate for each sample.

#### 3.4.4. X-ray Diffraction Analysis (XRD)

The X-ray diffraction spectra of the different samples were obtained, using an X-ray diffractometer (AXS/D8 Advance, Bruker, Karlsruhe, Germany), with Kα-Cu radiation (λ: 1.542 Å), at 40 kV, 40 mA, a step size of 2.0° min ^−1^, and scan angle 2θ ranging from 5° to 40°. The crystallinity index (CI), in percentage, was determined by applying Equation (2) [57] for each sample. This equation relates the maximum intensity of the 200-lattice diffraction (I_200_, crystalline peak) and the diffraction intensity of the amorphous phase valley at 2θ = 18° (I_2θ 18°_). Data acquisition was performed using XRD Commander software (version 8.61, Bruker AXS GmbH, Karlsruhe, Germany), and the results were processed using DIFFRAC.EVA and DRXWin (Windows, version 2.3).

(2) $$CI\left(\%\right)=\frac{\left(I_{200}-I_{2\theta 18{}^{\circ}}\right)}{I_{200}}\times 100$$

#### 3.4.5. Microstructure 

Morphological analyses of the raw material and the different lignocellulosic fractions obtained from the SWE and bleaching processes were carried out by field emission scanning electron microscopy (FESEM) (ULTRATM 55, Zeiss, Oxford Instruments, Abingon, UK). Conditioned samples (in P_2_O_5_ at 25 °C for one week) were coated with a platinum layer using an EM MED020 sputter coater (Leica BioSystems, Barcelona, Spain) for 60 s, and images were captured at an accelerating voltage of 1.7 kV.

## 4. Conclusions

Combining subcritical water extraction at 160 or 180 °C and subsequent bleaching with hydrogen peroxide (8%) at pH 12 allowed for obtaining cellulose from almond shell with good yields (30 and 26%, respectively, for samples treated at 160 and 180 °C) and cellulose purity (70 and 78%, respectively), with a reduced amount of chemicals. The highest extraction temperature promoted the removal of hemicellulose and subsequent delignification during bleaching. The use of sodium chlorite as a bleaching agent slightly improved the cellulose purification with a lower alteration of the cellulose structure. Indeed, a higher crystallinity index was obtained in the chlorite-bleached samples (69 and 64%, respectively, for samples treated at 160 and 180 °C). Therefore, almond shell could be used as a source of cellulose for different applications, using a more sustainable process, applying a previous extraction using only water under subcritical conditions. The use of hydrogen peroxide, as a greener bleaching agent, provoked small alterations in the cellulose particles of almond shell, but its properties can also be useful for different applications. Additionally, the first water extraction step allows for the recovery of potentially active compounds from the material, thus promoting a better exploitation of the biomass.

## Figures and Tables

**Table 1:** Content (g/100 g sample) of cellulose, hemicellulose, lignin, protein, and ashes of almond shell samples (ASs) and lignocellulosic residues obtained in SWE at 160 and 180 °C before (R160 and R180) and after bleaching treatments with hydrogen peroxide (BR160 and BR180) and sodium chlorite (BR160C and BR180C).

Sample	Cellulose	Hemicellulose	Lignin	Protein	Ashes
AS	26.8 ± 1.3 ^e^	23.6 ± 0.2 ^a^	21.2 ± 2.0 ^a^	4.7 ± 1.2 ^a^	2.0 ± 0.2 ^b^
R160	45.0 ± 0.1 ^c^	20.6 ± 0.7 ^b^	17.3 ± 0.2 ^b^	2.1 ± 0.4 ^b^	2.5 ± 0.1 ^b^
R180	41.7 ± 0.9 ^d^	11.6 ± 0.1 ^c^	18.6 ± 0.1 ^b^	1.9 ± 0.2 ^b^	2.1 ± 0.1 ^b^
BR160	70.5 ± 0.9 ^b^	20.3 ± 1.0 ^b^	8.5 ± 2.0 ^c^	nd	4.3 ± 0.3 ^a^
BR180	78.4 ± 0.2 ^a^	12.2 ± 1.2 ^c^	4.9 ± 1.2 ^d^	nd	4.1 ± 0.3 ^a^
BR160C	77.0 ± 4.0 ^a^	20.0 ± 2.0 ^b^	3.5 ± 0.5 ^e^	nd	1.7 ± 0.3 ^c^
BR180C	83.7 ± 2.4 ^a^	12.5 ± 0.7 ^c^	5.1 ± 0.6 ^d^	nd	1.1 ± 0.1 ^c^

Different letters in the same column indicate significant differences between films by the Tukey test (α = 0.05). Nd refers to non-determined values.

## Data Availability

The original contributions presented in the study are included in the article, further inquiries can be directed to the corresponding author.
